# The impact of new proposed arrhythmogenic right ventricular cardiomyopathy (ARVC) criteria on CMR diagnosis

**DOI:** 10.1186/1532-429X-15-S1-E117

**Published:** 2013-01-30

**Authors:** Luigi Natale, Agostino Meduri, Riccardo Marano, Lorenzo Bonomo

**Affiliations:** 1 Catholic University of Rome, Sesto Fiorentino (FI), Italy

## Background

In 2010, a modification of the 1994 Task Force Criteria for ARVC diagnosis was proposed.

CMR was officially introduced as a diagnostic tool, and quantification of RV functional parameters was added.

We evaluated the impact of new proposed CMR criteria in our previous series of patients with clinical and/or pathological diagnosis of ARVC.

## Methods

From January 2001 to December 2010, we studied 457 pts (279 males, 178 females; mean age 47±18 yrs) to exclude/confirm a clinical suspicion of ARVC; this suspicion was based on combination of clinical signs and symptoms, 12 leads-ECG data, anamnestic data, electrophysiologic data, and pathologic data, when available.

All CMR were performed with a clinical 1.5 T scanner (Signa Excite 2, GE Medical Systems), using a standardized protocol for cardiomyopathies (triple IR-black blood sequence, cine-SSFP on three axis optimized for RV, and late enhancement with 2D IR-FGRE).

## Results

Based on CMR data, we excluded 49 pts because of signs of previous infarcts, 103 pts because of signs of dilative primary or secondary cardiomyopathy, 38 because of signs of myocarditis, and 27 because of signs of hyperthrophic cardiomyopathy; in remaining 240 pts, RV regional function was evaluated and diameters, volumes and ejection fraction were calculated, 157 resulting normal and 89 abnormal.

Out of 89 abnormal RVs at CMR, 47 had a final diagnosis of ARVC, while out of 157 normal RVs, 6 had a final diagnosis of ARVC.

Looking at distribution of criteria, there were 52 major and 37 minor, with a significant modification compared to the original interpretation, that was: 192/240 abnormal CMR (76 with major criteria and 116 with minor), 48/240 normal CMR.

Significant decrease of major and minor criteria and increase of negative cases with new criteria was observed.

## Conclusions

One of the main aims of revisiting 1994 task force criteria was to improve sensitivity.

In our series, retrospectively evaluated, the application of new proposed MR criteria resulted in no changes in sensitivity and slight increase in specificity.

We guess prospective multicenter clinical trials to evaluate the effective role of new proposed criteria for ARVC diagnosis.

## Funding

Specificity and negative predictive value of MRI for ARVC were high with previous criteria and are slightly increased with new proposed one.

CMR is able to exclude with high confidence the disease.

